# Who’s Afraid of Adversariality? Conflict and Cooperation in Argumentation

**DOI:** 10.1007/s11245-020-09736-9

**Published:** 2020-12-23

**Authors:** Catarina Dutilh Novaes

**Affiliations:** 1 Department of Philosophy, VU Amsterdam, Amsterdam, The Netherlands; 2 Arché, University of St. Andrews, St. Andrews, Scotland, UK

**Keywords:** Adversariality, Cooperation, Argumentation, Agonism, Consensus, Scientific norms

## Abstract

Since at least the 1980s, the role of adversariality in argumentation has been extensively discussed within different domains. Prima facie, there seem to be two extreme positions on this issue: argumentation should (ideally at least) *never* be adversarial, as we should always aim for cooperative argumentative engagement; argumentation should be and in fact is *always* adversarial, given that adversariality (when suitably conceptualized) is an intrinsic property of argumentation. I here defend the view that specific instances of argumentation are (and should be) adversarial or cooperative *to different* degrees. What determines whether an argumentative situation should be primarily adversarial or primarily cooperative are contextual features and background conditions external to the argumentative situation itself, in particular the extent to which the parties involved have prior conflicting or else convergent interests. To further develop this claim, I consider three *teloi* that are frequently associated with argumentation: the epistemic *telos*, the consensus-building *telos*, and the conflict management *telos*. I start with a brief discussion of the concepts of adversariality, cooperation, and conflict in general. I then sketch the main lines of the debates in the recent literature on adversariality in argumentation. Next, I discuss the three *teloi* of argumentation listed above in turn, emphasizing the roles of adversariality and cooperation for each of them.

## Introduction

Since at least the 1980s, the role of adversariality in argumentation has been extensively discussed within different domains, including argumentation theory, feminist theory, critical thinking, deliberative democracy, and cognitive science. Some authors criticize adversarial conceptions and practices of argumentation, instead defending more cooperative approaches on both moral and epistemic grounds. Others retort that what is problematic is not adversariality per se, but rather overly aggressive manifestations thereof.

Prima facie, there seem to be two extreme positions on this issue: argumentation should *never* be adversarial, as we should always aim for cooperative argumentative engagement; argumentation should be and in fact is *always* adversarial, given that adversariality (when suitably conceptualized) is an intrinsic property of argumentation. Different authors who have contributed to these debates fall within a spectrum between these two extreme positions, some closer to the ‘cooperative’ extremity and others closer to the ‘adversarial’ extremity. In what follows, I defend the view that specific instances of argumentation are (and should be) adversarial or cooperative *to different degrees*; this is a descriptive as well as a normative/prescriptive claim. This view seems to fall right in the middle of the spectrum. What determines whether an argumentative situation should be primarily adversarial or primarily cooperative are contextual features and background conditions external to the argumentative situation itself, in particular the extent to which the parties involved have prior conflicting or else convergent interests.

To further develop this claim, I consider three *teloi* that are frequently associated with argumentation: the epistemic *telos*, the consensus-building *telos*, and the conflict management *telos*.^1^ (There may well be other presumed *teloi* for argumentation; this list is not intended to be exhaustive.) The epistemic *telos* consists in positing that the primary goal of argumentation is to promote epistemic improvement, understood as advancement of what we take to be valuable epistemic goals such as the attainment of knowledge and understanding, the maximization of true beliefs while minimizing false beliefs, etc. The consensus-building *telos* posits that the primary goal of argumentation is to lead to consensus whenever disagreement and dissent arises, in view of the need for social coordination in numerous circumstances. Finally, the conflict management *telos* highlights the role that argumentation can have in managing the inevitable conflicts that arise in any minimally complex human social setting. It is worth noting that I do not take any of these three *teloi* to be prima facie more fundamental than the others; one may be (as in fact I am) a pluralist about the goals of argumentation and maintain that argumentation may serve different purposes in different circumstances.^2^

In what follows, I discuss the extent to which (and under which circumstances) argumentation is indeed a suitable means to pursue these *teloi* by discussing different domains of human practices in which each of them seems to be applicable and/or relevant. In particular, for each of them I discuss when cooperative or else adversarial argumentation is the appropriate response (to different degrees, not necessarily as a binary opposition). This approach is intended to do justice to the multi-faceted nature of argumentation and to the insight that argumentative practices are embedded in complex social realities.

The paper proceeds as follows: I start with a brief discussion of the concepts of adversariality, cooperation, and conflict in general. I then sketch the main lines of the debates in the recent literature on adversariality in argumentation. Next, I discuss the three *teloi* of argumentation listed above in turn, emphasizing the roles of adversariality and cooperation for each of them.

## Adversariality, Cooperation, and Conflict

Let us start with some familiar but important observations. Humans are hyper-social animals; we need each other to survive. This means that there is pressure for humans to cooperate with conspecifics and care for each other. At the same time, as individuals we need to protect our interests and ensure access to essential resources for our survival; for this, we often need to compete with other humans. (Here there is a contrast with the social structure of other hyper-social animals such as bees and termites, where individuals routinely sacrifice their own lives to promote the wellbeing of the group as a whole.) Moreover, humans tend to create strong ties with specific groups, leading to intense within-group cooperation but competition with (and hostility toward) other groups and their individuals (in what is known as in-group/out-group dynamics (Ellemers and Haslam 2012)). The upshot is that human sociality is characterized by strong levels of cooperation *as well as* strong levels of competition and adversariality. Most theorists would agree with this observation, but there is substantive disagreement as to which of these two tendencies prevails: some emphasize the role of competition (e.g. Mercier and Sperber 2017), whereas others emphasize the role of cooperation in human sociality (e.g. Tomasello 2014). What is clear is that any explanation of human social behavior must take both phenomena into account.

Competition and adversariality are closely related but different concepts. Prima facie, competition presupposes *something* that is competed for by different parties, whereas adversariality may come about even if there is no specific good or resource that different parties wish to acquire but cannot have simultaneously. (See for example Casey’s conceptualization of adversariality in terms of involuntariness and attempts to control each other’s bodies and actions (Casey 2020).) For the present purposes, I adopt a conception of adversariality in terms of the *interests* that individuals or groups may have.^3^ Interests are states of affairs that individuals or groups wish to bring about; typically (though not necessarily), they will tend to contribute to the wellbeing of the individual or group. Some examples: it is in my personal interest to enjoy a rich network of friendships; it is in the interest of a given minority group in a society that they not be discriminated against in the relevant job market.

**Adversariality:** An individual or group A and another individual or group B are *adversaries* if (a) A has an interest *i*_*A*_ and B has an interest *i*_*B*_ such that *i*_*A*_ and *i*_*B*_ cannot simultaneously obtain, or the more *i*_*A*_ is satisfied, the less *i*_*B*_ is satisfied (and vice-versa), and (b) both pursue their own interests.

Notice that the implication runs only in one direction, as there might be other ways for A and B to be adversaries other than by having clashing interests. A special case of adversariality thus understood is when A and B are competing for some specific rivalrous good or resource which is scarce, such that A having more of it entails B having less of it (and vice-versa). But a formulation of the notion of adversariality in terms of interests provides a more general perspective that can also account for situations of adversariality where there is no obvious resource under dispute, but rather the obtainment of conflicting states of affairs.

Moreover, it is important to note that adversariality is distinct from egoism. On the one hand, it may be that A and B are not adversaries even though they only care about their own interests (and are thus egoists). This would happen if the promotion of A’s interests does not threaten the promotion of B’s interests. On the other hand, A and B may care about the wellbeing of others (Van Lange 1999) and yet still be adversaries (even if this might be a somewhat unusual situation). For example, Zollman (2020) shows that people can face difficult game-theoretic problems that effectively make them adversaries to others even if they altruistically pursue purely epistemic goals.

Furthermore, notice that the relation of adversariality between A and B obtains *relative to a pair of interests i*_*A*_ and *i*_*B*_. At least in principle, it is possible for A and B to be adversaries relative to *i*_*A*_ and *i*_*B*_, but allies relative to some other common interest (though the idea of ‘adversariality spillover’ seems plausible). But if there is a clash of interests thus described, then when A works towards promoting and enforcing *i*_*A*_, this will entail actions and interventions that will prevent *i*_*B*_ from coming about (partially or completely), thus obstructing B from obtaining the interests they pursue. Crucially, the relation of adversariality between A and B relies on actively pursued interests, not their idle interests, as specified by clause (b) in the definition above.

Cooperation between A and B, by contrast, may come about in different ways, such as^4^:

- *Egoistic cooperation (E-cooperation)*: *i*_*A*_ and *i*_*B*_ are sufficiently aligned so that A and B can cooperate while at the same time still primarily pursuing their own individual interests.
- *Joint cooperation (J-cooperation)*: A and B pursue a common interest which is best (or only) achieved by means of joint action. One might then say that A and B in fact form a new unit C and pursue *i*_*C*_ together.^5^
- *Altruistic cooperation (A-cooperation)*: A and B have conflicting interests *i*_*A*_ and *i*_*B*_, but A does not pursue these interests actively, thus leaving room for B’s interests to thrive. Instead, A actively promotes *i*_*B*_, despite it not being for her own individual benefit.^6^

In what follows, these conceptualizations will provide the theoretical background for an analysis of adversariality and cooperation specifically with respect to argumentation.

Before moving on to argumentation specifically, let me be explicit about a particular assumption I will be relying on: *conflict* is an inevitable and ineliminable component of human lives. Notice that the term ‘conflict’ is used here to refer to the quasi-ontological background of clashes of interests, whereas ‘adversariality’ is reserved for the actualization of conflict when different individuals or groups effectively pursue their conflicting interests. Here I follow authors such as Nietzsche (see Pearson 2018), Foucault (2003), and more recently Mouffe (2000) and Medina (2011) who hold a ‘tragic’ (in the ancient Greek sense) view of the human condition (Wenman 2013), and who have theorized about conflict and its political as well as epistemic implications. For these authors, conflict cannot be eliminated, but it can be channeled. Channeling and managing conflict is essential because conflict alone does not define human sociality; cooperation is just as central (Tomasello 2014). But given that different individuals and different groups will inevitably have interests that clash with those of others and will each pursue these conflicting interests to various degrees (at least this seems like a reasonable enough assumption), conflict seems to be a given in human sociality.

## Critiques and Defenses of Adversariality in Argumentation

Let us now turn specifically to argumentation. In recent decades, much has been written on adversariality in argumentation. In particular, a number of authors have argued against conceptualizations of argumentation (in philosophy as elsewhere) as inherently adversarial (Moulton 1983; Gilbert 1994; Cohen 1995; Rooney 2012; Hundleby 2013; Bailin and Battersby 2017). Many (but not all) of these authors formulated their criticism specifically from a feminist perspective. In turn, others have argued that adversariality, when suitably understood, can be seen as an integral and in fact desirable component of argumentation (Govier 1999; Aikin 2011; Casey 2020).^7^

Feminist critiques of adversariality challenge conceptions of argumentation as a form of competition, where masculine-coded values of aggression and violence prevail (Kidd 2020). For these authors, such conceptions encourage argumentative performances where excessive use of forcefulness is on display. These instances of aggressive argumentation in turn will have a number of problematic consequences: epistemic consequences—the pursuit of truth is not best served by adversarial argumentation—as well as moral/ethical/political consequences—these practices exclude a number of people from participating in argumentative encounters, namely those for whom displays of aggression do not constitute socially acceptable behavior (women and other socially disadvantaged groups in particular). These authors defend alternative conceptions of argumentation as a cooperative, nurturing activity (Gilbert 1994; Bailin and Battersby 2017), which are traditionally feminine-coded values. Crucially, they view adversarial conceptions of argumentation as *optional*, maintaining that the alternatives are equally legitimate and that cooperative conceptions should be adopted and practiced.

But what appears to be implicit in many (though not all of) these feminist analyses is a general rejection of *conflict* as such; emphasis on argumentation as a cooperative endeavor suggests that participants will straightforwardly have common interests, or else that conflict can be overcome through argumentation. More generally, the implication seems to be (though I am not aware of it being explicitly stated) that conflict as such, coded as masculine and with negative valence, is optional: it is bad, and it should be eliminated. In a post-patriarchal, utopian world, human sociality would be defined entirely by cooperative bonds. A weaker version of this position might be that conflict itself is not optional, but *enacting* conflict in argumentation is optional, and thus non-adversarial argumentation should be pursued.

These criticisms of adversarial argumentation have in turn been challenged in various ways. One overall theme is the need to draw a distinction between (excessive) aggressiveness and adversariality as such. Govier, for example, distinguishes between ancillary (negative) adversariality and minimal adversariality (Govier 1999). The thought is that, while the feminist critique of excessive aggression in argumentation is well taken, adversariality conceived and practiced in different ways need not have the detrimental consequences of more extreme versions of belligerent argumentation. More recently, Govier helpfully distinguishes between opposition to a *claim* and opposition to a *person* (Govier 2020). Moreover, for these authors, adversariality in argumentation is simply not optional: it is an intrinsic feature of argumentative practices. (But notice that Govier, Aikin and Casey each develop different accounts of adversariality in argumentation.)

From the conception of adversariality in terms of conflicting interests sketched in the previous section, it does not follow that argumentation is intrinsically adversarial.^8^ Indeed, imagine two arguers whose interests are sufficiently aligned (E-cooperation), or who share a common goal (J-cooperation); they may then engage in an exchange of reasons for the sake of pursuing these common interests (Norman 2016; Tomasello 2014). In such cases, there does not seem to be any obvious way in which the argumentative interaction will necessarily be adversarial, even if the arguers initially hold different opinions, and even if at the end of the interaction they still have not converged into a common view. Thus, adopting the perspective of adversariality as arising from conflicting interests, it seems perfectly possible for some argumentative encounters not to be adversarial at all, as claimed by the critics of adversariality. I also agree with them that, whenever possible and suitable, these kinds of cooperative argumentative encounters should be encouraged and promoted (for example in the context of education (Bailin and Battersby 2017)). Thus viewed, argumentation may become a joint action (J-cooperation) where the contribution of all parties is essential for the pursuit of the common interest in question—like pair dancing, duet singing, or pushing a car (on joint action, see Schweikard and Schmid 2013). In fact, the Lakatosian ‘proofs and refutations’ model of the production of mathematical knowledge (Lakatos 1976) is a quintessential example of argumentation as joint action, requiring the contribution of multiple parties to come about (as I argued in Dutilh Novaes (2020c), Chapter 11).^9^

However, I reject the (tacit) implication (or assumption) that conflict and adversariality in argumentation can (and should) be entirely eliminated. Even if there is a background of substantive interdependence and cooperation among humans, there will still be clashing interests that give rise to conflict and (insofar as actors pursue their respective interests at the expense of the interests of others) adversariality. In these situations, it may be perfectly appropriate for argumentation to be adversarial, for example in matters of social justice and political contestation. (More on this in Sect. 6; Aikin (2011) makes similar points.) In such cases, it is not argumentation that causes and gives rise to adversariality; adversariality already exists prior to the argumentative encounter, and argumentation may be one component of various strategies to manage (and potentially mitigate) adversariality and conflict. (Which is not to say that argumentation will always or even typically be the most adequate instrument to manage adversariality and conflict; more on this in Sect. 6.) Attempts to eliminate adversariality and manifestations thereof from argumentative encounters when there is already a background of conflict are not only futile, but also potentially dangerous: the conflict in question will be made invisible and left unacknowledged. What should be aimed at instead is what Aikin describes as *proportional adversariality* (see Appendix 1 for a simple model of how to measure the degree of adversariality between two agents).

The key is the maintenance of proportionality in exchange. Argumentative exchanges escalate sometimes justly and sometimes unjustly. It is necessary that we have at hand a variety of skills and techniques that mitigate harmful escalation. (Aikin 2011, p. 269)

In the next sections, I examine a number of situations where argumentative encounters occur, considering three of the presumed *teloi* of argumentation discussed in the literature. For each of them I discuss when cooperative or adversarial argumentation may be the appropriate response. Importantly, the cooperative vs. adversarial opposition is understood here as a matter of degrees rather than as a sharp distinction, hence the notion of proportional adversariality: the argumentative encounter may be adversarial to the extent that it reflects pre-existing levels of adversariality (and potentially other contextual factors as well). This means that a given argumentative situation may be *overly* adversarial, exacerbating or even creating conflict.^10^ But it may also be *insufficiently* adversarial, which may occur for example when the more powerful side of an interaction is in a position to suppress the justified objections and complaints by the less powerful (with devices such as ‘tone policing’ (Cherry 2018)), or when the less powerful do not feel sufficiently safe to speak up (Dotson 2014).

## Epistemic *Telos*

We speak of argumentation as having an epistemic *telos* when we take its primary purpose to be that of improving our doxastic position by increasing knowledge and understanding, and by helping us to maximize true beliefs and minimize false beliefs (as these can reasonably be taken to be our main epistemic aims). When critically examining reasons for and against a given position, we would be able to weed out the weaker, poorly justified beliefs (more likely to be false) and end up with stronger, suitably justified beliefs (more likely to be true). It is in this sense that argumentation is thought to be *truth-conducive*, at least in cases where there is an objectively correct answer to a problem.^11^ Goldman captures this idea in the following terms:

Norms of good argumentation are substantially dedicated to the promotion of truthful speech and the exposure of falsehood, whether intentional or unintentional. […] Norms of good argumentation are part of a practice to encourage the exchange of truths through sincere, non-negligent, and mutually corrective speech. (Goldman 1994, p. 30)

In this vein, a number of authors, most notably John Stuart Mill (and more recently Betz 2013; Mercier and Sperber 2017), maintain that interpersonal argumentative situations, involving people who truly disagree with each other, work best to realize the epistemic potential of argumentation. Mill famously defended this position in *On Liberty* (1859), positing that when our ideas are challenged by engagement with those who disagree with us, we are forced to consider our own beliefs more thoroughly and critically.

[Man] is capable of rectifying his mistakes, by discussion and experience. Not by experience alone. There must be discussion, to show how experience is to be interpreted. Wrong opinions and practices gradually yield to fact and argument; but facts and arguments, to produce any effect on the mind, must be brought before it. (Mill 1999, p. 41)

The expected result is that the remaining beliefs, those that have survived critical challenges, will be better grounded than those held before such encounters. As Mill puts it, “both teachers and learners go to sleep at their post, as soon as there is no enemy in the field.”^12^ (Mill 1999, p. 83). Dissenters thus force us to stay epistemically alert instead of becoming too comfortable with existing, entrenched beliefs. The presupposition seems to be, however, that even when disagreeing with each other, arguers involved in these encounters share a common goal, namely *epistemic improvement*, and that this common goal should overrule other tendencies such as seeking to ‘score points’ with an audience (‘arguing to win’ (Fisher and Keil 2016)). How realistic is this presupposition in real-life situations?

There is at least one domain of organized human activity where the epistemic *telos* of argumentation is presumed to prevail, namely *scientific investigation*. But even science is ultimately also a ‘human, all too human’ activity where factors such as competition and vanity also play a role. Indeed, let us start by acknowledging that, while ideally engaged in the common goal of advancing human knowledge, scientists also compete for scarce resources such as money to fund their research, visibility, reputation, jobs etc. In this sense, scientists can be viewed as *adversaries* of one another (in terms of the pursuit of conflicting interests). These competitive interactions can occur at group level (e.g. different groups working on cracking a particular scientific puzzle and competing to be the first one, given the significance of scientific priority (Strevens 2003)), or at individual level (e.g. different scientists competing for a given job). If scientists are understood as adversaries in this sense, it is not unreasonable to expect their argumentative practices to reflect this fact, such as being uncharitable when discussing opposing views, attacking straw-men, working on undermining the visibility of competing theories etc. As a descriptive claim, it is undeniable that scientists sometimes (often?) engage in overly adversarial argumentation, as attested by the long history of ugly scientific feuds (Levy 2010). The question remains whether it is *desirable* for adversariality to manifest itself in scientific practice and scientific argumentation in this way.

It might be thought that, even if individual scientists are exclusively (or predominantly) engaged in promoting their own interests rather than pursuing the goal of advancing human knowledge, as a whole the structure of scientific investigation still ensures that this epistemic goal is achieved. This would be the case precisely thanks to the fierce competition among scientists, which would ensure that only the strongest theories and ideas survive: individual vices would lead to a collectively virtuous system, a phenomenon known as ‘Mandevillian intelligence’ (Smart 2018).^13^ But it might also be that vices at the individual level (including practices such as scientific fraud, unfair competition, etc.) also end up corrupting science as a collective epistemic enterprise. If this is so, then there may be good reasons to manage and contain adversariality in scientific practice and argumentation.

Indeed, it may be argued that some of the very pillars of scientific practice and methods are intended to ensure that the epistemic *telos* be primarily pursued rather than the individual, non-epistemic interests of scientists (such as securing jobs, becoming famous etc.). The pioneer sociologist of science R. Merton famously described four sets of institutional imperatives (now known as ‘Mertonian norms’) that would comprise the ‘ethos’ of modern science (Merton 1942):

- *Communalism*: all scientists should have common ownership of scientific resources so as to promote collective collaboration.^14^
- *Universalism*: scientific validity is independent of the sociopolitical status or personal attributes of individual scientists.
- *Disinterestedness*: scientific institutions act for the benefit of a common scientific enterprise, rather than for the personal gain of individuals within the scientific community.
- *Organized skepticism*: scientific claims should be exposed to critical scrutiny by peers before being accepted.

These principles are presented as ideals that the scientific community as a whole should aspire to rather than as descriptive claims about actual scientific practice; Merton understood them to embody what most practicing scientists take to be the ethos of modern science.^15^ There has been much criticism of the Mertonian norms as a descriptive model and even as a normative model of scientific practice (Kim and Kim 2018). (The extent to which practicing scientists de facto adhere to them (both reflectively and in their practices) is itself an interesting empirical question.) But they can plausibly be seen as reasonable *regulative principles*: when followed, they should promote the epistemic *telos* of science and discourage the pursuit of non-epistemic individual interests. They offer a counterbalance to the fact that scientists are ultimately simply humans moved by their personal interests (as recognized by Merton himself (Kim and Kim 2018)). If scientific inquiry is still to be predominantly an epistemic enterprise, provisions must be in place to contain and suitably channel the pursuit of non-epistemic interests. Ideally, these principles should be enforced by means of institutional factors (including the education and training of future scientists) and with a suitable reward system.^16^

These principles also have interesting implications for argumentative practices in science, given that scientists also essentially engage in practices of ‘giving and asking for reasons’, seeking to persuade their peers of the cogency of their claims (Zamora Bonilla 2006). Communalism should ensure that the informational background of a scientific community is transparent, thus establishing sufficient common ground in these debates. Universalism entails the idea that what matters in science is the quality of the argument as such, not the reputation or standing of the scientist proposing it (although, as we know, this is far from descriptively accurate: epistemic injustices of various sorts routinely occur in science (Grasswick 2017)). Disinterestedness should ensure that, when engaging in a scientific debate, a scientist argues ‘to learn’ rather than ‘to win’ (Fisher and Keil 2016).

Finally, the principle of organized skepticism should have the effect of institutionalizing critical scrutiny so that scientific objections are not perceived as personal attacks (as is typically the case in more mundane dialogical interactions, which follow default norms of credulity (Aikin 2011, p. 268)). It is the *job* of a scientist to critically assess the arguments put forward by her peers, that is, to avoid being easily convinced. In this way, the scientist is in fact *collaborating* with peers when engaging critically, as objections and comments will help them improve their scientific theories. I describe in detail this form of ‘critical collaboration’ in mathematics in terms of a dialogue between two characters, Prover and Skeptic (thus echoing Mertonian organized skepticism) (Dutilh Novaes 2020c, Chapter 11), but the idea applies to scientific inquiry more generally.^17^

Organized skepticism thus understood does not in fact qualify as a form of adversariality in the sense adopted here: ideally, a scientist who is submitting the work of a colleague to critical scrutiny is pursuing the same interest as her colleague, namely to advance human knowledge.^18^ From this perspective, a scientist whose work is being criticized by a peer may be able to assuage the natural, instinctive reaction to become defensive when being criticized. Instead, she may, as Socrates, even come to *prefer* being refuted rather than to refute (Dutilh Novaes 2020b). The fact that, in practice, scientists will not always abide by this principle indicates that cultivating the argumentative virtue of being open to constructive criticism remains a challenge, also for scientists. Moreover, the reward system and institutions in science are far from perfect, sometimes encouraging practices of ‘scoring points’ in argumentation rather than the joint pursuit of knowledge. But at both levels (personal practices and institutions), offering and being open to constructive criticism remains a valuable ideal to aspire to.

Thus understood, it is natural to expect that there should be a fair amount of J-cooperation in science, that is, cooperation as joint action. The common goal of advancing human knowledge is arguably best pursued by collective and coordinated efforts by the scientific community as a whole; Charles Sanders Peirce was a famous proponent of this model of scientific practice (Nubiola 2014). (E-cooperation probably also has its place in science.) Admittedly, I have not categorically refuted the ‘Mandevillian intelligence’ account of scientific practice, but I take it to be much less plausible (both descriptively and prescriptively) than a joint action model, where scientists primarily and jointly pursue a common epistemic *telos* (but of course against a background of various forms of non-epistemic adversariality). Cooperative argumentation, including constructive criticism, is a key component of this model.

To conclude, in this section the epistemic *telos* of argumentation was discussed with a focus on scientific inquiry, which is arguably the organized human activity where this epistemic *telos* is most salient (for argumentation as well as more generally). The general points should apply to other domains where people share the common (primary) goal of advancing human knowledge as such (e.g. educational settings). The main point is that when such a convergence of interests occurs toward epistemic progress, it seems advisable to moderate and manage adversariality relative to other goals and interests, including in argumentative practices. Ideals of scientific integrity and respectful critical engagement can play the role of mitigating the interference of background non-epistemic conflicts of interests.

This being said, disagreement and dissent—which are here treated as distinct from adversariality, even so-called ‘minimal adversariality’^19^—can have a beneficial epistemic role insofar as they promote critical scrutiny. This point is aptly captured by the Mertonian norm of ‘organized skepticism’. Naturally, critical engagement can be weaponized and used in excessive forms (in philosophy in particular, as argued in Moulton (1983) and Rooney (2012)); but excluding criticism and dissent completely from science and intellectual inquiry in general is not desirable, hence the importance of ‘organized skepticism’. Instead, good scientific practices should ensure that criticism remains constructive, thus countering excessive adversarial tendencies that are not conducive to the epistemic *telos* of science.

## Consensus-Building *Telos*

Another important strand in the literature are theories that take *consensus* to be the fundamental *telos* of an argumentative process: to eliminate or resolve a difference of (expressed) opinion. The influential tradition of pragma-dialectic is one of the main exponents of this view: “argumentation has the general function of managing the resolution of disagreement.” (Van Eemeren and Grootendorst 1996, p. 278). Gilbert’s concept of coalescent argumentation is another approach that takes consensus to be the *telos* of argumentation (Gilbert 1997).

What seems to motivate these consensus-oriented approaches is the attribution of a role of *social coordination* to argumentation. Because humans are social animals and must often cooperate with other humans to successfully accomplish certain tasks, they must have mechanisms to align their beliefs and intentions, and subsequently their actions (Tomasello 2014). The thought is that argumentation would be a particularly suitable mechanism for such alignment, as an exchange of reasons would make it more likely that differences of opinion would decrease (Norman 2016). This may happen insofar as argumentation is indeed a good way to track truths and avoid falsehoods: by being involved in the same epistemic process of exchanging reasons, the participants in an argumentative situation can presumably converge towards the truth, and thus the upshot would be that they also come to agree with each other.^20^ However, consensus-oriented views need not presuppose that argumentation is truth-conducive: the ultimate goal of argumentation on these views is that of social coordination, and for this, tracking the truth is not a requirement per se, as long as beliefs, intentions and actions are aligned. This means that argumentation can lead to consensus also in matters where there isn’t necessarily one ‘truth’ to converge towards.

Indeed, the social complexity of human life is ultimately what motivates the emphasis on consensus. There are many important situations where some degree of consensus and coordination is necessarily, especially regarding *political decisions*. In particular, the very notion of deliberative democracy is viewed as resting crucially on argumentative practices (Landemore 2012; Fishkin 2016; Habermas 1996). (For the present purposes, ‘deliberation’ and ‘argumentation’ can be treated roughly as synonymous). Habermas, for example, defends the idea of the ‘public sphere’ as the space where deliberation aiming at a rational consensus takes place (Olson 2014). Political deliberation should allow for the collective organization of people’s lives, including the common rules we should live by. In a deliberative democracy, for a decision to be legitimate, it must be preceded by authentic public deliberation, not merely the aggregation of preferences that occurs in voting. When full consensus does not emerge, the different people involved may opt for a compromise solution. This is what usually happens in, for example, coalition-based political systems, where after an election typically a number of different parties must come together in a coalition to compose a majority government.

But how effective is argumentation when it comes to building consensus? The well-documented phenomenon of polarization (Isenberg 1986; Sunstein 2002) seems to suggest that argumentation is in fact not always a suitable means to reach consensus. Work on formal modeling of multi-agent argumentative encounters (Olsson 2013) offers further evidence suggesting that, when there is a certain amount of disagreement at the starting point and agents are given the opportunity to deliberate, they end up even further apart from each other in their opinions after engaging in argumentation.

Instead, it seems that it is only under quite specific circumstances that argumentation may lead to consensus, as also shown by the multi-agent simulations studied in Betz (2013). It is especially in situations of cooperation and alignment of interests that argumentation is likely to lead to consensus and to better solutions—in other words, situations of *collaborative decision-making*. Tomasello (2014) argues that exchanging reasons is a powerful tool in such situations; if the joint decision is to benefit all parties, then individuals want to make the best decision based on sound reasons and evidence (regardless of who ‘wins’ the argument), so they produce and evaluate reasons cooperatively as a means to that end. Tomasello and colleagues conducted a number of experiments that confirmed the efficacy of argumentation in cooperative settings, also showing that competitive settings may lead to suboptimal argumentative exchanges (with children at least) (Domberg et al. 2018).

By contrast, argumentation does not seem to be a particularly suitable means to reach consensus in situations of conflicts of interests, especially when the different parties pursue their clashing interests—in other words, when they are *adversaries* of each other, in the sense adopted here. Instead, what is more likely to ensue in such cases is polarization and further escalation of conflict (unless de-escalation measures are in place (Aikin 2011; Talisse 2019)).^21^

What is even more worrisome is that a focus on consensus may in fact end up reinforcing and perpetuating existing unequal power relations in a society. “In an unjust society, what purports to be a cooperative exchange of reasons really perpetuates patterns of oppression.” (Goodwin 2007, p. 77). Goodwin further argues that consensus-oriented accounts of argumentation, e.g. pragma-dialectic, seem to naively presuppose a homogeneous, harmonious society where deep conflicts of interests are not salient, and where (superficial) differences of opinion can be resolved in a ‘gentlemanly’ way through reasonable argumentation. This general point has been made eloquently by a number of feminist political thinkers (e.g. Young 2000), who have highlighted the exclusionary implications of consensus-oriented political deliberation *à la* Habermas (among others).^22^ The more ‘civilized’ and non-adversarial these discussions are expected to be, the more exclusionary they will be regarding those who have good reasons to be angry, and those whose communicative strategies do not fit the mold of what is considered reasonable, ‘polite’ discourse (Henning 2018).

In fact, the focus on consensus may be seen as an attempt to sweep the problem of conflict under the rug as it were, which, instead of solving it, is more likely to exacerbate it. This is a point often made by the theorists of *agonistic democracy*, who will be discussed in the next section. The gist of their criticism can be thus summarized:

Where liberals and deliberative democrats typically seek to overcome or transcend conflict by bringing it under a set of regulative principles (foundational principles of justice or context-transcending principles of communicative rationality), the agonists insist that these responses actually serve to exacerbate the problem. Instead, we should look to sublimate this hostility by transforming it into more constructive modes of rivalry. (Wenman 2013, p. xiii)

In other words, conflict in a society cannot be made to disappear by simply ‘wishing it away’, that is, by postulating that consensus can always (or even typically) be achieved. Reasonable or rational argumentation will not by itself, contrary to what many seem to think, reliably lead to the resolution of disagreements, in particular when the interests of the different parties are not aligned, or when they do not share fundamental values.^23^ (We will return to these points in the next section.)

In sum, consensus-oriented argumentation seems particularly suitable in cooperative settings, especially in cases of J-cooperation, where the joint decision is to benefit all involved parties. In adversarial settings however, not only is argumentation not likely to resolve (in the sense of giving rise to consensus) disagreements that stem from deeper conflicts of interests. Under the naïve assumption of a shared interest—to reach consensus—consensus-oriented argumentation may in fact end up reinforcing patterns of oppression. This does not mean, however, that argumentation has no place in situations of (political) adversariality; we will see in the next section that argumentation can be an instrument to *manage* (instead of resolving) conflict and disagreement.

## Conflict Management *Telos*

So let us now turn to the conflict management *telos* of argumentation. In a sense, the consensus-building *telos* as previously discussed is a special case of conflict management, based on the assumption that the best way to manage conflict and disagreement is to aim for consensus. But conflict can be managed in different ways, not all of them leading to consensus; indeed, some authors maintain that argumentation may help mitigate conflict even when the explicit aim is not that of reaching consensus (as we will see).^24^ To this category also belong the conceptualizations of argumentation-as-war discussed (and criticized) by a number of authors (Cohen 1995; Bailin and Battersby 2017); in such cases, conflict is not so much managed but rather *enacted* (and possibly exacerbated). Thus seen, the *telos* of argumentation would not be fundamentally different from the *telos* of other organized competitive activities such as sports or even war (with suitable rules of engagement) (Aikin 2011).

Importantly, authors who identify conflict management (or variations thereof) as the *telos* of argumentation differ in their overall appreciation of the value of argumentation: some take it to be at best futile and at worst destructive,^25^ while others attribute a more positive role to argumentation in conflict management. In what follows, we focus on these more optimistic accounts, in particular on the concept of *agonism* as developed by Chantal Mouffe.

Let us start with the observation that, when conflict with others emerges, people have a variety of options on how to manage the situation: they may choose not to engage and flee instead (maybe because the reason for conflict is not worth the risks involved); they may go into full-blown fighting mode, which may include physical aggression; or they may opt for approaches somewhere in between the fight-or-flight extremes of the spectrum. Argumentation would belong to this intermediary category, as described by Aikin:

[A]rgument literally is a form of pacifism—we are using words instead of swords to settle our disputes. With argument, we settle our disputes in ways that are most respectful of those who disagree—we do not buy them off, we do not threaten them, and we do not beat them into submission. Instead, we give them reasons that bear on the truth or falsity of their beliefs. However adversarial argument may be, it isn’t bombing. […] argument is a pacifistic replacement for truly violent solutions to disagreements… (Aikin 2011, p. 256)

This is not to say that argumentation will always or even typically be the best approach to handle conflict and disagreement; the point is rather that argumentation at least has the potential to do so, provided that the background conditions are suitable and that provisions to mitigate escalation are in place (Aikin 2011). Versions of this view can be found in the work of agonistic authors, in particular (but not exclusively) Chantal Mouffe, for whom democratic practices, including argumentation/deliberation, have the function to contain hostility and transform it into more constructive forms of contest (while also recognizing the inevitability of conflict). In the same vein, Mouffe offers compelling criticism of consensus-oriented theories of deliberative democracy such as those of Habermas and Rawls (Mouffe 1999).

While theories of agonistic democracy differ substantially from each other (Wenman 2013), they all seem committed to three basic tenets:

(i) an emphasis on constitutive pluralism, (ii) a tragic vision of a world without hope of final redemption from conflict, suffering, and strife, and (iii) a belief that certain forms of contest can be a political good. (Wenman 2013, p. 18)

The agonist thus posits that pluralism is at the heart of the human experience, which is constituted by a plurality of perspectives, views, ways of life, values etc. Pluralism is not just the starting point to be overcome by means of (argumentative or otherwise) consensus-forming procedures; instead, it is an ineliminable and in fact *desirable* feature of social realities, in particular given our aspirations towards freedom. But where there is pluralism, there is conflict, as these different ways of life will typically not be content with simply coexisting side by side. In particular, on many occasions individuals and groups will become *adversaries* of each other in the sense that they pursue clashing interests, hence the tragedy of perennial conflict and strife. However, at least some forms of conflict and contestation are essential for the healthy political life of a society: “a democratic polity, conflicts and confrontations, far from being a sign of imperfection, indicate that democracy is alive and inhabited by pluralism.” (Mouffe 2000, p. 34).

Among the different agonistic authors (conveniently surveyed in Wenman 2013), Mouffe in particular attributes to democratic institutions the crucial role of transforming (or ‘sublimating’ in the Freudian sense, as she prefers to put it) hostility and aggression into constructive forms of conflict and contest. To conceptualize these processes, Mouffe relies on two related distinctions: ‘the political’, which is understood in terms of *antagonism*, and ‘politics’, which is understood in terms of *agonism*:

I have developed these reflections on ‘the political’, understood as the antagonistic dimension which is inherent to all human societies. To that effect, I have proposed the distinction between ‘the political’ and ‘politics’. ‘The political’ refers to this dimension of antagonism which can take many forms and can emerge in diverse social relations. It is a dimension that can never be eradicated. ‘Politics’, on the other hand, refers to the ensemble of practices, discourses and institutions that seeks to establish a certain order and to organize human coexistence in conditions which are always potentially conflicting, since they are affected by the dimension of ‘the political’. (Mouffe 2013, p. 2/3)

Conflict in liberal democratic societies cannot and should not be eradicated, since the specificity of pluralist democracy is precisely the recognition and the legitimation of conflict. What liberal democratic politics requires is that the others are not seen as enemies to be destroyed, but as adversaries whose ideas might be fought, even fiercely, but whose right to defend those ideas is not to be questioned. To put it in another way, what is important is that conflict does not take the form of an ‘antagonism’ (struggle between enemies) but the form of an ‘agonism’ (struggle between adversaries). (Mouffe 2013, p. 7)

While Mouffe does not discuss argumentation specifically, it seems reasonable to read her reference to “the ensemble of practices, discourses and institutions” that constitutes liberal democracies as including argumentation and deliberation. Argumentation should thus play an important role in the sublimation of antagonism into agonism. Notice however that this is an ongoing process, not an end-point: antagonism remains in the background and rears its ugly head time and again.^26^ In fact, Mouffe maintains that denying the perennial existence of conflict and antagonism, which she describes as “the typical liberal gesture,”^27^ is particularly dangerous:

Firstly, the predominant democratic praxis is in denial about the reality of ‘the political’ and, secondly, and ironically perhaps, this naive renunciation actually exacerbates conflict and makes antagonism more likely, because these tendencies open the door to extremist parties who claim to offer a meaningful alternative to mainstream consensus elites. In other words, the emphasis on consensus provokes a ‘return of the political’ in the form of a heightened potential for antagonism… (Wenman 2013, p. 181)

Indeed, Mouffe credits the rise of extreme-right populism in the last 20 years to the excessive focus on consensus and disregard for the ever-present underlying antagonistic forces in any society. “Antagonistic conflicts are less likely to emerge as long as agonistic legitimate political channels for dissenting voices exist. Otherwise dissent tends to take violent forms.” (Mouffe 2005, p. 21).

Notice that the claim is not that, by itself, argumentation will be able to tame and ‘sublimate’ antagonism into agonism. Even at the level of discursive practices, other forms of communicative engagement appear to be required to foster a truly inclusive public sphere of discourse. The political theorist Marion Iris Young (who was not an agonist thinker herself) in particular argued that *greeting* (understood as public acknowledgment), *rhetoric*, and *narrative* are important communicative actions that come to complement argumentation/deliberation so as to ensure inclusiveness (Young 2000, Chapter 2). But with suitable provisions to counter excessive escalation in place (Aikin 2011), argumentation may constitute a useful tool to manage conflict and channel hostility into constructive forms of conflict: turning enemies into adversaries, as Mouffe puts it. We are here reminded of how various forms of martial arts (and sport competitions more generally) may be seen as attempts to channel aggression and hostility towards constructive, virtuous adversariality (see Kidd (2020) on the analogy between martial arts and practices of argumentation). However, the goal should not be to suppress conflict entirely; this is not only impossible, but in fact also perilous in that, paradoxically, suppression attempts increase the likelihood of violent manifestations of dissent and conflict.

It may be thought that these considerations only apply to political aspects of argumentation, having no bearing on the epistemic *telos* discussed above. However, conflict in a pluralistic society can also have beneficial epistemic consequences. In this vein, Medina (2011, 2013) transposes the value of agonism to the epistemic domain through the concepts of *epistemic friction* and *guerilla pluralism*:

The epistemic friction produced by the interaction of heterogeneous standpoints can yield a critical awareness of multiple ways of perceiving and can point in the direction of change, of the melioration of our perceptual attitudes and habits. (Medina 2013, p. 224)

[Guerilla pluralism] is not a pluralism that tries to resolve conflicts and overcome struggles, but instead tries to provoke them and to re-energize them. It is a pluralism that aims not at the melioration of the cognitive and ethical lives of all, but rather, at the (epistemic and socio-political) *resistance* of some against the oppression of others. (Medina 2011, p. 24)

More generally, the point is that argumentation that takes the form of resistance and contestation in contexts of social injustice and oppression should indeed be adversarial, in the sense of containing vigorous critiques of the status quo. Attempts to eliminate conflict from argumentation entirely may in fact end up *favoring* the status quo, a conclusion that is surprisingly at odds with the feminist critique of adversariality in argumentation. Of course, adversarial argumentation can also be used as an instrument of oppression, and the feminist critique of argumentative adversariality as a form of exclusion must be taken seriously. So a rule of thumb might be that the more powerful side of an argumentative encounter should generally speaking be less ‘adversarial’, whereas the less powerful side may avail themselves of more forceful modes of argumentative intervention: after all, there can be no social change without irritating the powerful.

## Conclusion

In this paper, I formulated a conception of adversariality in terms of conflicting interests so as to discuss the role of adversariality in argumentation. Overall, the paper presents a defense of *proportional adversariality*: an argumentative situation should be adversarial in proportion to the pre-argumentative levels of adversariality (conflict of interests) between the parties involved. In cooperative settings, that is, when people share common goals and may join forces to achieve them together, adversariality in argumentation is unnecessary and often counterproductive. By contrast, if there is an antecedent background of conflict of interests, then it may well be desirable and appropriate to engage in adversarial, forceful argumentation. I take it that conflict as such is an inevitable feature of human sociality, and attempts to ‘wish conflict away’ often do more harm than good. What is required instead are ways to channel and manage conflict; with suitable provisions in place to contain harmful escalation, argumentation can be one of the strategies used for this purpose.

## Appendix 1: How to Measure Adversariality (Joint Work with Hein Duijf)

The notion of proportional adversariality seems to require a way to ‘measure’ more or less precisely the degree of adversariality between two parties. So here is a simple formal model of how this could be done. The model is obviously an idealization far removed from complex real-life situations, but it indicates that the notion of proportional adversariality need not be hopelessly vague and imprecise.

Consider two agents (or groups), A and B. Each has a set of interests pertaining to states of affairs S_1_, S_2_, S_3_, S_4_ … Interest I_Ak_ represents A’s position with respect to state of affairs S_k_, which is one of three options:

- I_Ak_ = 1: A wants S_k_ to obtain,
- I_Ak_ = −1: A wants S_k_ not to obtain,
- I_Ak_ = 0: A is indifferent with respect to S_k_.

A’s interests generate an ordered set of numerical values I_A_ = (I_A1_, I_A2_, I_A3_ … I_Az_), and the same applies to B’s interests: I_B_ = (I_B1_, I_B2_, I_B3_ … I_Bz_). The *degree of interest alignment* between A and B can be determined by comparing I_A_ and I_B_ in terms of the distance between each of the paired values in the sets. So if I_Ak_ = 1 and I_Bk_ = −1, the distance corresponds to 2; if I_Ak_ = 1 and I_Bk_ = 0, the distance corresponds to 1; if I_Ak_ = 1 and I_Bk_ = 1, the distance corresponds to 0 etc. Adding up the scores for each comparison pair I_Ak_ and I_Bk_ yields a numerical value on a scale from full conflict (2*z*, where *z* is the index of the last item in each interest set I_A_ and I_B_) to full alignment (0).

## Footnotes

1 I follow Stevens and Cohen (2019) when using the *telos* terminology. Notice that these *teloi* are understood as capturing the (presumed) structural functions of argumentation as a whole, not the personal motivations that individuals have to engage in argumentation (though these two can of course overlap to some extent). So for example, one might articulate the view that individuals join argumentative encounters purely to win the argument and ‘score points’, but that as a whole argumentative practices still serve the overall function of improving the epistemic state of a community or of leading to consensus.

2 But see Goodwin (2007), who argues compellingly that approaching argumentation in terms of function and *telos* raises a host of difficult issues.

3 Casey (2020) draws a distinction between adversariality pertaining to interests and adversariality as control, and then goes on to focus on the latter; here I focus on the former. However, this does not necessarily mean that there is significant disagreement between us. For the purpose of philosophical theorizing, we have a certain amount of freedom in how to understand and define certain concepts; the main guiding principle is that of *fruitfulness*, in the spirit of Carnapian explication (Dutilh Novaes 2020a). My choice here is to focus on interests as a defining feature of adversariality so as to (hopefully) shed some new light on the issues under discussion, thus complementing Casey’s analysis.

4 This list is not intended to be exhaustive.

5 See the plural subject theory in Gilbert (1990) and the team reasoning account of cooperation in Gold and Sugden (2007).

6 For example, a feminist man may work toward promoting the interests of women in his society even if this may mean he will personally lose some of the male privileges he enjoyed so far. (Such people are described in activist circles as ‘allies’.) Notice that here cooperation will typically (though not necessarily) be asymmetric: A cooperates with B, but not the other way round. A-cooperation will not feature prominently in what follows, as it is not immediately clear how instances of A-cooperation can be relevant specifically for argumentative practices. But one might think of *learning contexts*, where A engages in argumentation with B so as to increase B’s understanding of a given topic, even if this entails an expenditure of time and energy for A that may have no immediate concrete pay-off for her (other than the satisfaction of helping B).

7 To be sure, there are important differences between the authors in each of these two groups, which for reasons of space cannot be properly addressed.

8 It may well be that, if adversariality is conceptualized differently, argumentation will still come out as intrinsically adversarial. So I am not contesting the intrinsicality claim in general, but only with respect to this particular conceptualization of adversariality in terms of conflicting interests.

9 However, one may retort that, while two agents may collaborate to construct an argument together, the argument in turn may be intended for another agent off scene, a non-present adversary. (I owe this point to Scott Aikin.)

10 This observation reflects the recognition that argumentation by itself can become a catalyst for adversariality and hostility. As noted by Aikin (2011, p. 286), an argumentative interaction has a number of structural features that make it prone to escalation, such as its potential to cause irritation and its departure from default conversational norms of credulity. See also Dutilh Novaes (2020b) on the irritation and unpleasantness that can be caused by argumentation.

11 It is known from the literature on group problem-solving (surveyed in Laughlin 2011) that, for intellective problems, that is, those that have a unique answer within a given theoretical framework (e.g. a mathematical or logical problem), group discussion has a clear beneficial, truth-conducive effect. But for so-called judgmental problems, that is problems for which there is no unique solution and judgments of values are involved, the evidence is less clear.

12 Notice here an instance of the familiar ‘argument-as-war’ metaphor (Lakoff and Johnson 1980; Cohen 1995).

13 “I’ve heard the pragma-dialecticians say just this: the more hostile arguers are—the more they want to defeat each other—the more they are motivated to produce high quality arguments, and thus the more functional their joint activity becomes.” (Goodwin 2007, p. 79).

14 Merton’s communalism is closely related to the ‘principle of publicity’ (Piccinini 2003).

15 An important caveat here is that much of what is now done under the heading of ‘scientific research’ is aimed at specific applications funded by interested parties, e.g. the pharmaceutical industry. The Mertonian norms would thus apply primarily to what is often referred to as basic, foundational research, not to industrial research. Since the 1990s, there has been increasing commercialisation of academic science, which is clearly in tension with the classical Mertonian norms (especially communalism and disinterestedness) (Kim and Kim 2018).

16 Unfortunately, it is far from obvious that the current reward system in science is suitable in this way. For example, Bright (2017) has argued for the surprising conclusion that pursuing the epistemic, veritistic *telos* in science may in fact encourage vicious behavior such as fraud. “It turns out that a sincere desire on the part of scientists to see the truth propagated may well promote more fraud rather than less.” (p. 291).

17 See Stevens and Cohen (2020) for an articulation of the view that critical engagement—playing ‘Devil’s advocate’—can significantly contribute to argumentation’s goals.

18 Similarly, the protocol of ‘adversarial collaboration’ for empirical research, which consists in two researchers/groups who have opposite views on a given scientific open question or controversy teaming up to investigate their topic of disagreement (Mellers et al. 2001; Matzke et al. 2015), does not count as adversarial in the sense countenanced here. The scientists have different views, but they (presumably) share the common goal of advancing human knowledge. What this protocol shows is the epistemic value of *dissent*, provided that there be sufficient alignment of interests and goals.

19 This is the main reason why, in my work on deductive arguments, I moved away from the ‘proponent-opponent’ terminology, which suggests adversariality, towards the ‘Prover-Skeptic’ terminology (Dutilh Novaes 2020c).

20 Again, what the literature on group decision-making suggests is that argumentation and deliberation is truth-conducive in particular for intellective problems (Laughlin 2011), and assuming common goals/interests. The same does not seem to be straightforwardly the case in other situations (judgmental problems and/or conflict of interests).

21 One example of Aikin’s de-escalation measures is to ensure that argumentative encounters can begin with and be moderated by the gestures of personal connection. See also Young’s communicative actions to mitigate exclusion from debates discussed in the next section.

22 See also Geuss (2019) on the limitations of the Habermasian framework in real-life political situations.

23 Isaiah Berlin’s notion of *value pluralism* is relevant here: “In Berlin’s terms, competing values are often (but not always) ‘incommensurable’ and ‘there might exist no single universal overarching standard that would enable a man to choose rationally between’ them.” (Wenman 2013, p. 30). That is, disagreement about these values cannot be resolved by means of rational argumentation. These would be instances of what is now known as *deep disagreements* (Fogelin 1985).

24 Of course, a minimal amount of cooperation and alignment of interests is required to recognize the need (or desire) to manage and control conflict rather than let it blow up. But it can of course happen that particular political strands in a society in fact seek to let conflict blow up as a way to acquire or maintain power.

25 Some examples of pessimistic assessments of the value of argumentation would be Schopenhauer’s satirical account of ‘dialectic’ in *Eristic Dialectic: The Art of Winning an Argument*, and Schmitt’s bleak appreciation of parliamentary debates: “The essence of liberalism is negotiation, a cautious half-measure, in the hope that the definitive dispute, the decisive bloody battle, can be transformed into a parliamentary debate and permit the decision to be suspended forever in an everlasting discussion.” (Schmitt 2005, p. 63).

26 “In Mouffe’s view, every consensus appears as a contingent ‘stabilisation of something essentially unstable and chaotic’, and this constitutive instability should not be seen – with Hegel, Habermas, or the contemporary theorists of deliberative democracy – as a ‘temporary obstacle… on the road… towards harmony and reconciliation’.” (Wenman 2013, p. 195).

27 “The political in its antagonistic dimension cannot be made to disappear by simply denying it or wishing it away. This is the typical liberal gesture, and such negation only leads to the impotence that characterizes liberal thought when confronted with the emergence of antagonisms and forms of violence that, according to its theory, belong to a bygone age when reason had not yet managed to control the supposedly archaic passions.” (Mouffe 2013, p. 3/4).
